# The effect of shame on prosocial behavior tendency toward a stranger

**DOI:** 10.1186/s40359-022-01021-1

**Published:** 2022-12-15

**Authors:** Saiqi Li, Liusheng Wang

**Affiliations:** 1 College of Marxism, Nanjing University of Science and Technology, Nanjing, People’s Republic of China; 2 Teachers College, Jimei University, 185 Yinjiang RD., Jimei District, Xiamen, 361021 People’s Republic of China

**Keywords:** Public shame, Private shame, Prosocial behavior tendency, Stranger, Benefactor, Motive of shame

## Abstract

**Background:**

This study aimed to examine the effect of different types of shame on prosocial behavior tendency to different help-seekers.

**Methods:**

A total of 120 participants were randomly assigned to a neutral mood condition, a public shame or a private shame condition.

**Results:**

All participants rated their willingness to help a benefactor and a stranger in an everyday helping situation and a money-donating situation after emotion-induction. The study found a higher willingness of participants in the public shame group to help strangers than those in neutral mood and private shame groups.

**Conclusion:**

These findings support a facilitation effect of public shame on prosocial behavior tendency toward strangers, indicating an effect of restoring motive of shame on social interaction. The results are further discussed in light of the functionalism of shame.

## Introduction

Shame is one of the self-conscious emotions which arises from self-relevant failures and transgressions [1]. Based on this perspective, de Hooge [2, 3] proposed that a restore motive of shame affirms a positive self, while a protect motive of shame serves to avoid further damage to the self, indicating a specific role of shame in self. Regarding the protect motive, Sznycer et al. [4] reported that shame induces individuals to perform moral behavior to eliminate or attenuate their negative feelings, and to defend against a devaluation from others. Lewis [5] also viewed a threatened or damaged self as the central focus of experiences of shame. Concerning the restore motive, shame could encourage individuals to consider others’ interests, drive them to take corresponding prosocial behaviors, leading to increasing their well-being and improving social relationships [6, 7]. Similarly, according to Commitment Device Theory [8], selfishness may tempt people to choose immediate rewards at the expense of others, which creates unpleasant moral feelings, such as shame. Shame serves as a commitment device by raising the costs of selfish behavior, making immediate selfish options less attractive, motivating individuals to act in a prosocial manner and thereby committing them with long-term prosocial strategies. Further, the functionalism of shame focuses on social adaptation, viewing shame as a product of psychological evolution [8].

To date, the literature is not uniform about the positive effect of shame on prosocial behavior [7, 9–11], partly due to the definition of shame. Shame is an affective reaction to public exposure or disapproval of a transgression [12, 13]. Shame is part of a family of emotions including embarrassment, humiliation, shyness, failure, and incompetence, linked by threats to the social contract. It goes hand in hand with humiliation and embarrassment, but shame is not necessarily a bad thing. De Hooge et al. [3] found that individuals experiencing either endogenous (relevant to a decision at hand) or exogenous (not relevant to a decision at hand) shame preferred to be together with others over being alone, indicating the similar promoting effects of both types of shame on social approach. Public shame in particular is defined as the degree to which the general public holds negative views and discriminates against a specific group and the public know our shame experience [14]. The direct person whose we interact don’t know our shame experience, which is termed as private shame. Therefore, the difference between the two types of shame is that I or others know the shame experience. These are possibly related, but not entirely overlapping types of shame compared to private and public shame. Different shame types demonstrate different imparts on different positive behaviors in different studies. Therefore, the roles of shames in processing prosocial behavior remain unclear.

Besides the help-givers’ emotions, prosocial behavior is affected by various other factors, such as the roles of help-seekers or the relationships between help-seeker and help-giver. According to the reciprocal altruism theory, prosocial behavior in social interactions entails reciprocal altruism [15], or direct reciprocity [16], especially in Chinese culture, which addresses a harmonious atmosphere. An old Chinese proverb also says, “One good turn deserves another”. Being good to the benefactor is a social norm and a basic rule in a modern civilized society. Reciprocity and reciprocal altruism are found in several species, including human beings and animals, and even rats [17]. Being an altruist becomes the most helpful strategy in social interaction, which could be explained by natural selection [18]. Triver’s theory states that individual is sensitive to prosocial behavior, resulting in keeping a balance in the local social and ecological environment and benefiting each other in the environment. Even where there is no reciprocity, one might do a favor for somebody in a society. Actors who help strangers are judged to be more moral than actors who help their kin [19]. This could be understood in the context of in-group bias. In-group favoritism means one person shows more positive attitudes toward the in-group than the out-group [20], even though the members from the in-group are strangers to him.

For this study, public shame referred to situations where one’s prior shameful experience was known by another person who is seeking help, while private shame referred to situations in which the help-seeker had no such awareness. When a help-seeing stranger who without awareness of the person’s prior shameful experience seeks help, shame would be expected to activate the person’s restore motive. The restore motive of shame implicates shame as commitment device [2, 3, 6], encourages the individual to create and keep a better impression and to act prosocially. However, when facing a help-seeker who is a prior benefactor, an individual will act by reciprocal altruism. Thus, the research question of this study is: what is the influence of public versus private shame on prosocial behavior tendency toward a benefactor versus stranger?

In order to answer the research question, a 3 × 2 mixed experiment was designed with shame types (public/private/control) and the roles of help-seeker (benefactor/stranger), with type of shame as a between-subject variable and role of help seeker as a within-subjects variable, and willingness to help others and reaction time to decide to help or not as dependent variables. Accordingly, following shame functionalism, we proposed the following hypothesis. *Hypothesis* one: Public shame would be related to higher willingness to help. *Hypothesis* two: Individuals are more willingness to help a benefactor than a stranger.

## Methods

### Participants

A total of 120 college students from a eastern university were paid to participate in the experiment and were randomly assigned to neutral mood group, public shame group, or private shame group. Participants of all the groups together were aged *M* = 20.34 (*SD* = 2.08), 62 were male. Forty-one participants were in a neutral mood condition (control condition), aged *M* = 21.37, *SD* = 1.97, 37 in the public shame condition, aged *M* = 19.46, *SD* = 2.02, and 42 in the private shame condition, aged *M* = 20.12, *SD* = 1.85. The ratio of gender in each group was similar. A pre-study was conducted by additional 60 college students who evaluated the emotion-induction materials. All participants reported no history of mental disorder and had normal or corrected-to-normal vision. Participants were told that an experiment was about emotion-induction, and signed the informed content.

### Materials

The neutral mood and shame induction materials were adapted from everyday events in Chinese culture [21]. Sixty college students rated the intensity of shame on a 7-point scale after listening to the relaxing music “*Dancing with the Neon Light*” for 2 min, which had participants in a stable mood. The participants were required to imagine themselves as the protagonist in different situations. Three situations were selected as neutral mood materials, including an introduction to campus, a product manual, and an introduction to a music category. Three situations were selected as shameful experiences, and the themes included failure in an exam, littering in public, and failing to answer questions in class. To test three materials, three prompts of shame-inducing materials were given before shame was self-rated.

The materials for prosocial situations were adopted from helping events [22]. For example, an everyday helping situation was about lending some learning materials to others; a money-donating situation was about donating money to person suffering after an earthquake. Participants were faced with a help-seeker who was described as either a benefactor who had helped the participants or a stranger who never met the participants.

### Procedure

This experiment consisted of a shame-induction stage and helping-others stage. All participants listened to the same soothing music (“*Dancing with the Neon Light*”) for one minute in a laboratory room with a laptop computer before the experiment, which had participants in a stable mood. Participants were then randomly assigned to the neutral mood group, the public shame group, or the private shame group.

**Fig. 1:**
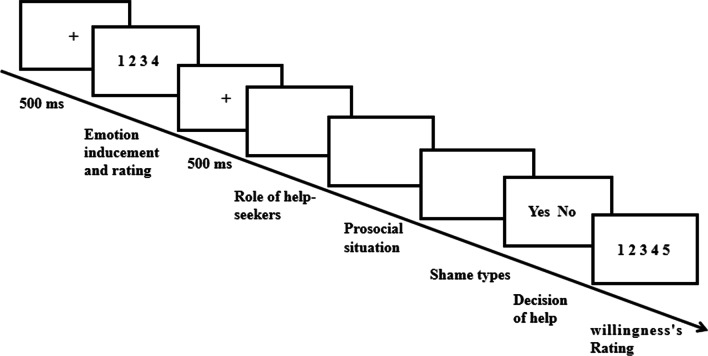
The Procedure of experiment

After the instruction, a red fixation “+” was present on the center of a computer screen for 500 ms, followed by the emotion-inducing written material (see Fig. 1). Participants in the neutral mood group read neutral materials, while participants in public and private shame groups read shame materials, imagined themselves as the protagonist in the stories, and rated their own intensity of shame on a 4-point scale (1–4); the bigger number referred to the stronger intensity shame. After a red fixation was present for 500 ms, participants were told a help-seeker was either a benefactor who helped them in two prior prosocial situations involving everyday helping and donating money, or a stranger who had never previously met them. Then two helping-seeking requests were read, the order of which was alternated. Under these two situations, the benefactor or stranger sought general help or money-donating from the participants. After this, participants were told that the help-seeker knew about a prior shameful experience involving the participant (public shame group) or did not know it (private shame group). Participants were then required to choose “Yes” or “No” to for helping or not and the participants’ reaction time, in which the participants’ reaction time to make a decision to help or not (decision-time) were recorded. Finally, participants rated their degree of willingness to help the help-seeker on a 5-point scale (1–5). After the experiment, each participant was debriefed.

## Results

As regards the test of materials, the ratings of shame one three kinds of neutral mood materials were 1.58 (*SD* = 1.15), 1.60 (*SD* = 1.07), and 1.62 (*SD* = 1.05), respectively. The average ratings of shame for three kinds of shame-inducing materials were 6.18 (*SD* = 1.05), 5.85 (*SD* = 1.00), and 4.61 (*SD* = 1.61), respectively. The *t*-tests of ratings found significant differences (*ps* < 0.05) between neutral and shame materials.

Analysis of variance (ANOVA) was conducted on the ratings of shame intensity. The results showed a significant difference among the three groups, *F* (2, 117) = 196.39, *p* < 0.001, η^2^ = 0.77. Post-hoc tests found the ratings of the neutral mood group (*M* = 1.19, *SD* = 0.31) were significantly smaller than those of the public (*M* = 3.11, *SD* = 0.51) and private shame groups (*M* = 2.97, *SD* = 0.59), and there was no significant difference between the public and private shame groups, indicating a high validity of the emotion-induction.

### Analysis of everyday helping behavior

**Fig. 2:**
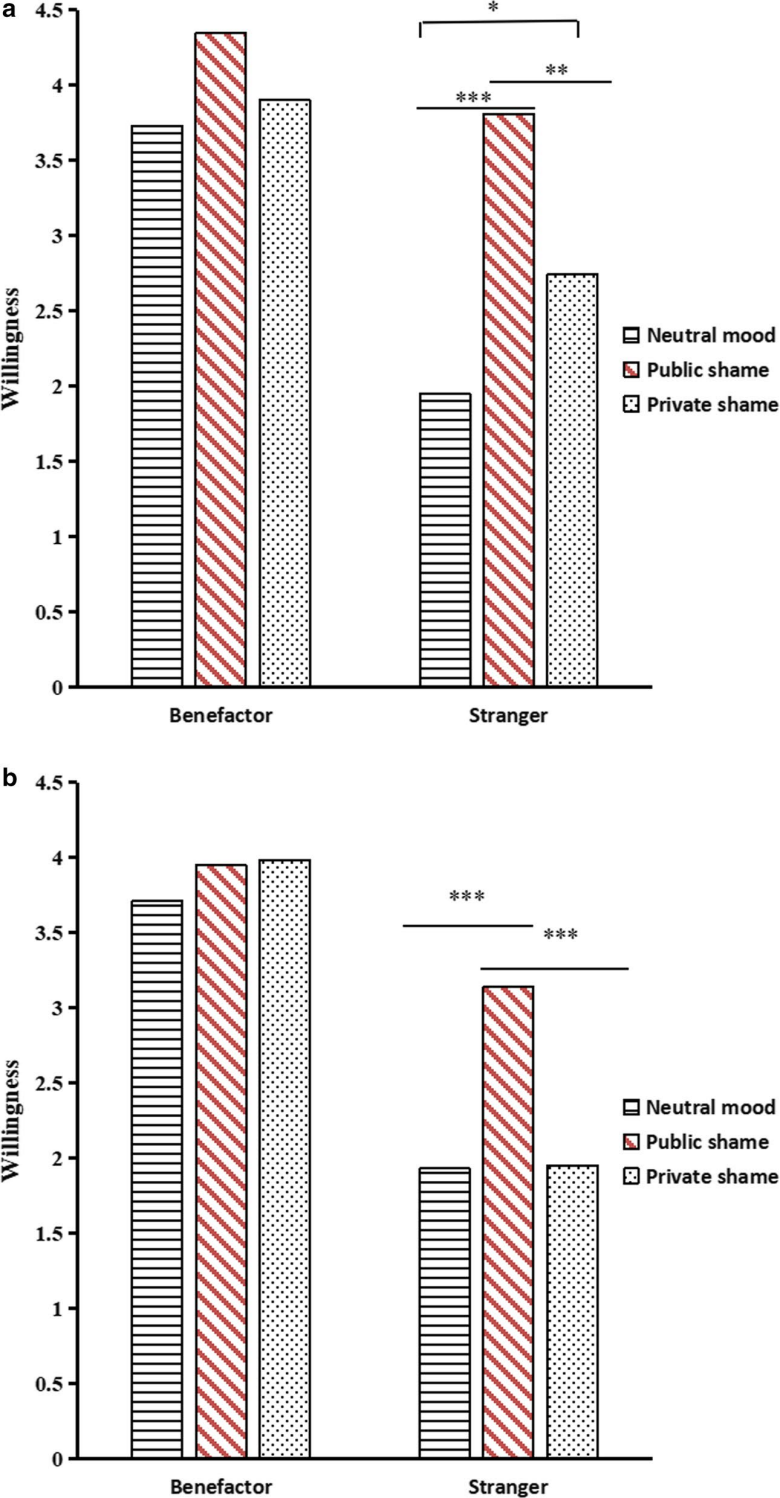
The interaction effect between shame types and roles of help-seekers on willingness for everyday helping (**a**) and donating money (**b**). *p < 0.05, **p < 0.01, ***p < 0.001

A repeated-measures ANOVA on willingness of everyday helping showed a significant main effect of shame types, *F* (2, 117) = 12.64, *p* < 0.001, η^2^ = 0.18, with willingness of everyday helping in the public shame group (*M* = 4.05, *SE* = 0.18) being significantly higher than that in the neutral mood (*M* = 2.84, *SE* = 0.17) and private shame groups (*M* = 3.32, *SE* = 0.17), and higher in the private shame group than that in the neutral mood. The main effect of help-seekers’ roles also was significant, *F* (1, 117) = 63.65, *p* < 0.001, η^2^ = 0.35, with willingness to help the benefactor (*M* = 3.97, *SD* = 1.20) being significantly higher than willingness to help the stranger (*M* = 2.80, *SD* = 1.62)*.* A significant interaction of shame types and help-seekers’ role was found, *F* (2, 117) = 6.61, *p* < 0.01, η^2^ = 0.10. Simple effects analysis showed no significant difference between shame types when participants were benefactors, *F* (2, 117) = 2.88, *p* > 0.05. In contrast, participants in the public shame group were more willing to perform everyday helping than those in the neutral mood and private shame groups, and participants in the private shame group were more willing to perform everyday helping than those in the neutral mood group when help-seekers were strangers (see Table 1, Fig. 2a), *F* (2, 117) = 16.07, *p* < 0.001, η^2^ = 0.22.

**Table 1:** Willingness and decision-time of participants in different conditions

Shame types	Help-seekers’ roles	Willingness of everyday helping		Decision time of everyday helping (ms)		Willingness of donation		Decision time of donation (ms)	
		*M*	*SD*	*M*	*SD*	*M*	*SD*	*M*	*SD*
Neutral mood	Benefactor	3.73	1.50	6007.32	4584.78	3.71	1.35	6915.07	3757.84
	Stranger	1.95	1.53	6987.56	5652.09	1.93	1.54	8085.88	5472.42
Public shame	Benefactor	4.30	0.85	6975.81	6162.39	3.81	1.29	8338.70	5864.31
	Stranger	3.81	1.24	7586.00	7873.87	3.16	1.07	7132.14	6130.81
Private shame	Benefactor	3.91	1.10	6105.24	4877.75	3.98	0.95	6338.67	3591.95
	Stranger	2.74	1.53	6487.41	5328.96	1.95	1.46	6622.86	4594.41

### Analysis of donating money

A repeated-measures ANOVA on willingness to donate money showed a significant main effect of shame types, *F* (2, 117) = 4.49, *p* < 0.05, η^2^ = 0.07. Willingness to donate money in the public shame group (*M* = 3.49, *SE* = 0.17) was significantly higher compared to the neutral mood (*M* = 2.82, *SE* = 0.16) and private shames groups (*M* = 2.96, *SE* = 0.16). The main effect of help-seekers’ roles also was significant, *F* (1, 117) = 106.19, *p* < 0.001, η^2^ = 0.48. Willingness to donate money to the benefactor (*M* = 3.83, *SD* = 1.20) was significantly higher than willingness to donate to the stranger (*M* = 2.32, *SD* = 1.48). The interaction of shame types and help-seekers’ role was significant, *F* (2, 117) = 8.37, *p* < 0.001, η^2^ = 0.13. A simple effects analysis found no significant difference between shame types, *F* (2, 117) = 0.53, *p* > 0.05; while participants in the public shame group were more willing to donate money than were those in the neutral mood and private shame groups, when help-seekers were strangers (see Table 1, Fig. 2b), *F* (2, 117) = 10.00, *p* < 0.001, η^2^ = 0.15.

## Discussion

Our objective was to determine whether or not shame types could have positive effect on prosocial behavior tendency toward a benefactor or a stranger in prosocial situations. This study found a promoting effect of public shame on prosocial behavior tendency toward strangers. Specifically, individuals in the public shame condition demonstrated a stronger willingness to help a benefactor who had ever helped the participants, rather than a stranger. Thus, the Hypothesis one was supported, which supports the functionalism of shame.

As expected, the help-seeker being a benefactor and the public shame condition were both related to higher levels of willingness to help. The finding of the positive effect of public shame on prosocial behavior tendency toward a stranger is somewhat similar to the findings by de Hooge et al. [7]. They found that endogenous shame (i. e. related to a decision at hand) motivated prosocial behavior for ‘proselfs’ (people with a natural tendency to act selfishly) but that exogenous shame (not related to a decision at hand) did not. Our study focused on a prosocial behavior tendency towards strangers in a social environment, while de Hooge and her colleagues [7] focused on behaviors of ‘proselfs’ or ‘prosocials’ in dilemmas. Nevertheless, the present findings are not in line with a later study showing the similar effect of endogenous and exogenous shames on social approach [3]. The social approach studied by de Hooge et al. [3] is not identical to the everyday helping and donating money in our study. The former focuses on the Withdrawal/Approach choice. The latter refers to the direct help to others and closer to the definition of prosocial behavior. Moreover, the effect of private shame on prosocial behavior was not at the cost of reaction time in the present study, meeting the requirement of speed-accuracy trade-off. Different shame presents different imparts on positive behavior in different prosocial situations, primarily depending on the contribution of a restore motive of shame.

The finding supporting the Hypothesis one could be explained by the functionalism of shame. Fessler [23] suggested that there are two major social functions of shame expression, namely to seek approval from others and to prevent social exclusion. Shame stems from the exposure of defects. Through the actual evaluation of others or the internalization of others’ evaluations, self-evaluation ultimately triggers shame [4]. A person cares about others' opinions and comments on his own behavior. And shame is more likely to arise from the presence of others [13]. Therefore, shame leads people to pay more attention to their positive image and good reputation in the eyes of others. Shame results in threats to social self-image [24]. In order to maintain a positive self-image, individuals take actions to repair their damaged self, such as limiting the spread of negative information about themselves and preventing the resulting devaluation or negative evaluation of others [3], which provides a possible explanation for shame promoting prosocial behavior. When violating the rules of the community and expressing shame, one is ready to conform to the group’s standards. In addition, this indicates to others that one is still a potentially valuable cooperation partner and a fully intergraded member of the group. At this time, one is more willing to behave in accordance with the rules of society. The display of shame has been shown to reduce punitive intentions by increasing the perpetrator’s moral sense, and evokes empathy in the observers, which further reduces the punitive intentions [25].

Compared with public shame, private shame means the defects’ exposure to others is prevented. The help-seeker in prosocial situations knows the individual is going through shameful experiences under the public shame condition in the current study. When a shameful experience is exposed to a partner in interpersonal interaction, it triggers a much stronger restore motive to adopt complementary strategies, such as prosocial behavior, to affirm a positive self-image. Especially in China, social ethics which place particular emphasis on the importance of protecting the public honor of one’s family and community acts as a central framework of social control, encouraging the masking of shameful private behavior with a public veneer of conformity. As long as shame experiences are concealed from the public gaze they can be tolerated. This framework of honor and shame is intrinsically linked to a public–private binary, which restricts the performance of all but normative identities through the constant repetition and restatement of acceptable identities [26]. Perceptions of public shame held by society are uniquely related to having positive attitudes toward seeking help. Individuals are likely to experience both positive and negative messages from those close to them, thus public shame may be more pervasive and represent clearer positive messages about seeking help. Furthermore, over time the public shame that society places on help is bound to change for the positive as well. Prosocial behavior is an optimal way for a person experiencing shame to restore the damaged self, to create a better impression and keep it in public, or to protect his/her damaged self from further damage [27].

Most importantly, as expected in the Hypothesis two, the present study provides a novel finding that the enhanced effect of public shame on prosocial behavior tendency came out only when help-seeker was a stranger. The roles of help-seekers in prosocial situations, a benefactor or a stranger, have different implications for people experiencing shame. Prosocial behavior is more often taken as repayment and reciprocity if an individual experiencing public shame helps a benefactor compared to helping a stranger. This prosocial behavior highlights an obligation in Chinese culture stressing harmonious interpersonal relationships, abiding by the basic rule “reciprocal altruism” in interpersonal communication [15–17], leading to a relative weakening of the restorative effect of shame. On the other hand, prosocial behavior toward a stranger is a better way to eliminate the negative feeling, affirm a positive self, implement the restored motive of shame. When the individual is under the condition of private shame, namely, the shameful experience is not exposed to a stranger, they have no risk to be devalued or despised by the stranger. Under this circumstance, one could maintain a positive self in the eyes of a stranger and more easily behave with kindness to the stranger.

The results also demonstrated a similar pattern of private shame on prosocial behavior tendency towards strangers in everyday helping situation and donating situation. As usual, donating money costs a person more than everyday helping because of cost–benefit analyses [28]. Donating money indeed means a lot for children, as indicated by Wang et al. [11]. However, it does not mean the same to adults who are in the current study. Donating pocket money does not mean a heavy loss for college students and it would not affect their everyday lives. Therefore, in a prosocial situation, whether it be an everyday helping situation or a donating situation, is not an influential factor for adults’ prosocial behavior.

Although this study has yielded some encouraging findings, some limitations still need to be noticed. We set the willingness to help others as an index of prosocial behavior tendency in this study, to measure the influences of emotion on behavior. However, one’s willingness might be different from the actual behavior in the real situation. Therefore, future studies should design a real situation that allows researchers to measure participants’ prosocial behavior. Guilt and shame both have important moral functions, and they are important factors that affect individual's subsequent positive social behavior. Therefore, future studies should design shame and guilt to measure prosocial behavior.

In conclusion, the current study demonstrated that public shame affects prosocial behavior tendency to a stranger. These findings cast light on the relationship between shame and positive behavior, and provide some implications in social interaction.

## Data Availability

All data generated during this study are included in this published article and its supplementary information files.
